# FTY720 Effects on Inflammation and Liver Damage in a Rat Model of Renal Ischemia-Reperfusion Injury

**DOI:** 10.1155/2019/3496836

**Published:** 2019-03-20

**Authors:** Anthony D. Foster, Diego Vicente, Nicholas Clark, Crystal Leonhardt, Eric A. Elster, Thomas A. Davis, Matthew J. Bradley

**Affiliations:** ^1^Department of Regenerative Medicine, Naval Medical Research Center, Silver Spring, Maryland, USA; ^2^Department of Surgery, Uniformed Services University and Walter Reed National Military Medical Center, Bethesda, Maryland, USA

## Abstract

**Objective:**

Ischemia-reperfusion injury (IRI) produces systemic inflammation with the potential for causing organ failure in tissues peripheral to the initial site of injury. We speculate that treatment strategies that dampen inflammation may be therapeutically beneficial to either the initial site of injury or peripheral organs. To test this, we evaluated the impact of FTY720-induced sequestration of circulating mature lymphocytes on renal IRI and secondary organ injury.

**Methods:**

A microvascular clamp was surgically placed around the left renal pedicle of anesthetized male Sprague-Dawley rats with either vehicle or FTY720 treatment (0.3 mg/kg) intravenously injected after 15 min of ischemia. Blood flow was restored after 60 min. Cohorts of anesthetized rats were euthanized at 6, 24, or 72 hrs with tissue samples collected for analysis.

**Results:**

FTY720 treatment resulted in profound T lymphocyte reduction in peripheral blood. Histopathologic examination, clinical chemistries, and gene transcript expression measurements revealed that FTY720 treatment reduced hepatocellular degeneration, reduced serum markers of liver injury (ALT/AST), and reduced the expression of gene targets associated with IRI.

**Conclusion:**

These findings support an anti-inflammatory effect of FTY720 in the liver where the expression of genes associated with apoptosis, chemotaxis, and the AP-1 transcription factor was reduced. Findings presented here provide the basis for future studies evaluating FTY720 as a potential therapeutic agent to treat complications resulting from renal IRI.

## 1. Introduction

Renal ischemia-reperfusion injury (IRI) can result from both transplantation and pathophysiologic processes such as massive hemorrhage and resuscitation or from the development and treatment of abdominal compartment syndrome. The subsequent oxidative stress response from renal tissue hypoxia and reperfusion injury can not only cause acute kidney injury (AKI) but can also lead to significant downstream sequelae in other organs in the body [1, 2]. That is, loss of blood flow to the kidney(s) and subsequent reperfusion pose a risk not only to the primary affected tissue but to peripheral organs as well.

At the tissue level, prolonged ischemia can produce considerable cellular damage and eventual necrosis that in turn facilitates the expression, activation, and nuclear translocation of key proinflammatory mediators, cytokines, chemokines, adhesion molecules, and reactive oxygen species (ROS) [1]. Hypoxic stress initiates an inflammatory cascade through mechanisms that are not yet fully understood. Initially, ischemic tissue damage results in the release of danger-associated molecular patterns (DAMPs) [3] which stimulate the innate immune system to produce inflammatory cytokines that increase vascular permeability, recruit additional leukocytes to the site of inflammation, and exacerbate tissue damage [4, 5]. Following reperfusion of the ischemic tissue, these mediators are released systemically where they may promote an inflammatory cascade leading to the systemic inflammatory response syndrome (SIRS). Ultimately, this inflammatory response can result in local and distant (peripheral) tissue injury with infiltration of neutrophils, activation of the endothelium, and release of ROS leading to organ dysfunction beyond the initial ischemic insult. In fact, it has been argued that focusing only on the renal aspects of AKI has not been sufficient, and greater efforts to protect distant organs following traumatic injury are necessary [6]. The implications of AKI to overall physiology should be understood then as a complex interaction between impaired renal function and distant organs. Importantly, inflammation plays a critical role in driving pathogenesis after traumatic injury such that greater inflammatory cytokine expression is associated with significantly higher mortality [7, 8].

Lymphocytes, including T cells, play an active role in mediating the severity of IRI in multiple animal models [9, 10]. The use of animals with severe combined immunodeficiency (SCID) for IRI studies, which do not generate T cells, results in a reduced severity of local injury to ischemic tissues [10, 11]. The removal of CD4^+^ T cells, but not CD8^+^ T cells, has been shown to reduce the severity of injury in mouse models of IRI [11]. Similarly, T cell-specific inhibition of NF-*κ*B in a murine model of renal IRI improved kidney function while reducing pathologic damage compared with control animals [12]. Blockade of CD40 : CD40L (CD154) signaling using anti-CD40 si-RNA in a rat model of warm renal IRI was likewise found to improve kidney function while reducing T cell-specific TNF-*α* production [13]. While T cells are a major component of adaptive immune responses, they appear also to play a role in the innate response to hypoxia [9]. As such, treatments that either suppress T cell function or limit availability may mitigate IRI.

Recently, we have demonstrated that FTY720, a sphingolipid immune modulator that interacts with the sphingosine-1-phosphatereceptor to sequester lymphocytes in lymphoid tissues, effectively reduces inflammation in a rat model of tourniquet-induced hind limb ischemia [14]. Likewise, FTY720 has been shown by others to mitigate the severity of hemorrhagic shock [15] as well as reduce the severity of lung IRI [16]. FTY720 has successfully been used to reduce injury in a mouse model of renal IRI [17, 18]. However, several features of these models make them difficult to interpret with respect to the value of FTY720 as an agent to be given following traumatic injury. First, the mouse models used by Okusa et al. included two doses of FTY720 given 24 and 20 hours prior to surgical injury. While this approach is highly relevant to models of transplantation, they are much less relevant to IRI resulting from trauma, where pretreatment is not possible. Second, the studies cited used ischemia times of 32 minutes and 24 minutes, respectively [17, 18]. The goal of the study presented here was to reflect the so called “golden hour” of therapeutic intervention, within 1 hour of injury. As such, a longer period of renal ischemia followed by reperfusion would be more clinically relevant to IRI resulting from trauma. While the mechanism of FTY720 intervention in these studies remains unclear, the sequestration of T cells in lymphoid tissues may result in decreased inflammation at the site of injury [19, 20]. It is also possible that FTY720 may alter neutrophil trafficking [1, 21] and modulate endothelial response to trauma. With that in mind, the goal of our study was to investigate the potential therapeutic impact of FTY720 treatment, given after ischemic injury, on both local (ischemic kidney) and peripheral tissues using a rat model of renal IRI.

## 2. Methods

### 2.1. Animals

Young adult pathogen-free male Sprague-Dawley rats (*Rattus norvegicus*; 300-350 g) were used for this experiment (Taconic Farms, Germantown, NY). All animals were housed individually in plastic cages and kept on a 12-hour light/dark cycle with unlimited access to food (standard rodent chow) and fresh water ad libitum. They were acclimated for at least one week before experimentation. The study protocol was reviewed and approved by the Walter Reed Army Institute of Research/Naval Medical Research Center Institutional Animal Care and Use Committee in compliance with all applicable federal regulations governing the protection of animals in research.

### 2.2. Renal Ischemia-Reperfusion Injury Model

Prior to experimentation, 10 rats were randomly assigned to serve as naïve (uninjured) controls. The remainder of the rats were randomized into either a vehicle control or FTY720 treatment group and assigned for euthanasia at 6, 24, or 72 hours (hrs) post-IRI (*n* = 10 for each survival time point). All control and FTY720-treated rats received an initial intraperitoneal (i.p.) injection of Telazol (40 mg/kg) to achieve adequate anesthesia. To induce renal IRI, a midline laparotomy incision was made under sterile conditions and a microvascular clamp was placed around the left renal pedicle. Visual inspection of the kidney was used to confirm blanching and cessation of blood flow. The total clamp time was 60 min, after which the clamp was removed to restore the blood flow into the kidney. Due to limited solubility in saline, FTY720 was diluted in ethanol first, then combined with saline to achieve a 50% ethanol solution. FTY720 (0.3 mg/kg) or vehicle (50% ethanol plus saline) treatment was administered as a single bolus 15 min after renal clamping (0.75 mL via the lateral tail vein).

### 2.3. Euthanasia, Blood Collection, and Tissue Harvest

Anesthetized rats in each group were euthanized using Fatal-Plus Solution (0.5 mL i.p; Vortech Pharmaceuticals, Dearborn, MI) followed by exsanguination via cardiac puncture. Blood was collected in EDTA tubes for total WBC, lymphocyte, and neutrophil analysis (CBC) with differential (CHEM-7, ALT, alkaline phosphate, creatinine, and albumin) and polychromatic flow cytometric analysis of T cell subsets. The spleen, lungs, ischemic and nonischemic kidneys, and liver were collected and processed as described below.

### 2.4. Pathologic Analysis of Histology

**Figure 1:**
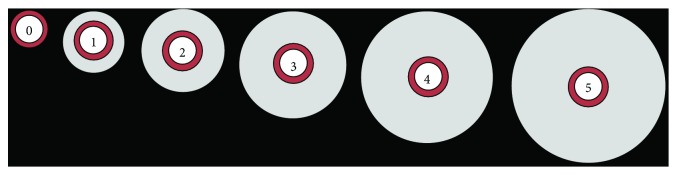
Graphic representation of perivascular edema scores 0 through 5. The white inner circle represents the vascular lumen, the red ring represents the arterial wall, and the gray outer circle represents the extent of perivascular edema surrounding the vessel.

Tissue samples were fixed in 10% formalin, paraffin-embedded, sectioned, and stained with hematoxylin and eosin. Samples were evaluated and scored by a veterinary pathologist, blinded to the protocol, using the scoring criteria described in Tables 1 and 2 and Figure 1.

### 2.5. RNA Isolation and Gene Expression

At the indicated time points, small samples of the lung, liver, ischemic kidney, and nonischemic kidney were harvested and stored in RNAlater (Ambion Inc., Austin, TX, USA) at 4°C. Tissue samples obtained from age-matched naïve uninjured rats (*n* = 6) served as control tissue. Total RNA was isolated and purified using RNeasy columns and DNase-I kits (Qiagen, Valencia, CA, USA) according to the manufacturer's protocols. RNA was stored at –80°C in nuclease-free water (Bio-Rad, Hercules, CA, USA). Total RNA was quantified spectroscopically by using NanoDrop 1000 (Thermo Fisher Scientific, Waltham, MA), and RNA integrity/quality was assessed by microcapillary electrophoresis using an Agilent 2100 Bioanalyzer (Agilent Technologies, Santa Clara, CA). Reverse transcriptase polymerase chain reaction (RT-PCR) was used to synthesize cDNA from 1 *μ*g of total RNA. mRNA transcripts for 86 key ischemia-reperfusion-related target genes were examined by real-time PCR (QuantStudio 7 Flex Real-Time PCR System; Applied Biosystems, Foster City, CA) using a custom low-density microarray (RT^2^ Profiler PCR array, Qiagen). Gene expression was normalized to a reference gene (GAPDH) and calculated relative to that of tissues collected from noninjured rats using the 2^-ΔΔCt^ method [22].

### 2.6. Flow Cytometry

Flow cytometry analysis was used to quantify T cells in circulation and in the spleen by CD45, *αβ*-TCR, CD4, and CD8 surface marker expression. Approximately one-third of each spleen was gently crushed using a sterile syringe head in staining buffer (PBS+1% FCS+0.01% NaN_3_) and filtered through a 70 *μ*m nylon cell strainer (Falcon, Tewksbury, MA). Isolated splenocytes were washed and resuspended in staining buffer. Cell staining was performed using standard methods. Briefly, 10^6^ splenocytes or 100 *μ*L of whole blood was pretreated for 10 min with Fc Block (100 *μ*L in staining buffer containing 0.5 *μ*g of anti-rat CD32 (BD Biosciences, San Jose, CA)) to prevent nonspecific Fc receptor-mediated antibody binding. Cells were first stained with saturating concentrations of anti-CD45-APC-Cy7 (BioLegend, San Diego, CA), anti-CD4-V450 (BD Biosciences, San Jose, CA), anti-*αβ*-PerCP (BD Biosciences,), and anti-CD8-PE-Cy7 (eBioscience, San Diego, CA) for 30 min at 4°C in the dark. Next, whole blood samples were lysed for 3 min at room temperature using ammonium chloride lysis buffer (ACK; Lonza, Walkersville, MD). All samples were washed twice and resuspended in staining buffer. Subsequently, all samples were fixed/permeabilized and stained intracellularly with anti-FoxP3-APC (eBioscience), washed, and then fixed in 1% paraformaldehyde following the instruction in the intracellular staining kit provided by the manufacturer (eBioscience). Samples were analyzed with a BD FACSAria™ II cell sorter and BD FACSDiva™ software (Becton, Dickinson and Company, San Jose, CA) using forward versus side scatter gating.

### 2.7. Statistical Analysis

This study has numerous categorical (e.g., histological grading) and continuous outcomes of interest (e.g., serum creatinine, cellular counts, and cell surface marker assessments). Continuous outcomes were compared between control and treatment groups at each time point using Student's two-tailed *t*-test, while categorical outcomes were compared using Mann–Whitney tests. *p* values of ≤0.05 (two-sided) were considered statistically significant. Data are represented as mean ± SEM.

## 3. Results

### 3.1. FTY720 Treatment Effectively Sequesters T Lymphocytes in Renal Ischemia-Reperfusion Injury

**Figure 2:**
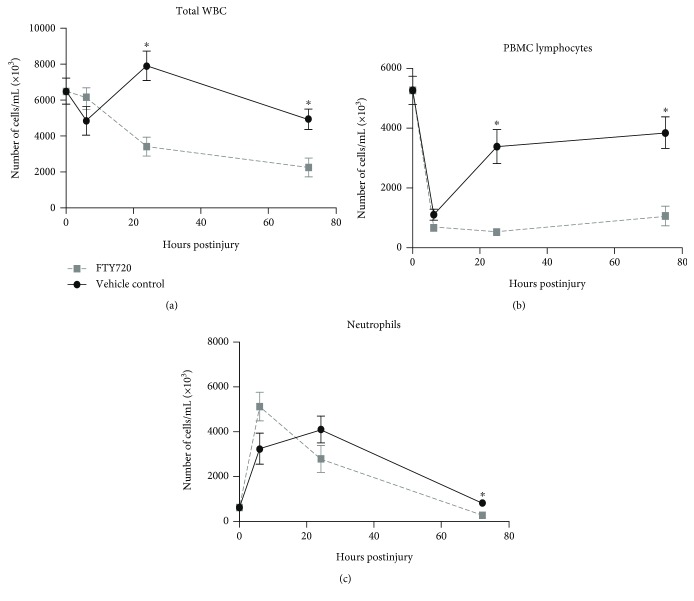
FTY720 treatment alters kinetics of peripheral leukocytes following renal ischemia. Rats underwent renal ischemia by the surgical placement of a microvascular clamp around the left renal pedicle of the anesthetized rat. Rats received a single intravenous bolus of either vehicle control (black circle) or 0.3 mg/kg FTY720 (gray square) 15 min after placement of the clamp. Vehicle control and FTY720 groups were euthanized at 6, 24, or 72 hrs. Peripheral blood CBC analysis was performed on blood collected by cardiac puncture. Data are shown as the average number of cells per mL for each group ± SEM (*n* = 6-10 rats per time point/group). ^∗^ *p* < 0.05 compared with vehicle control at the same time point postinjury.

The induction of unilateral renal ischemia followed by reperfusion after 60 min resulted in transient alterations in the number and type of circulating leukocytes. Total white blood cell (WBC) count in vehicle control animals modestly increased 22% above naïve control levels (6.48 × 10^6^/mL) at 24 hrs (7.89 × 10^6^/mL) (Figure 2(a)). The effect of FTY720 treatment on leukocytes was most apparent at 24 hrs postinjury. Total WBCs decreased by 47% at 24 hrs (3.42 × 10^6^/mL) and 65% at 72 hrs (2.27 × 10^6^/mL) with respect to naïve controls. Circulating lymphocytes (Figure 2(b)) decreased by approximately 80% at 6 hrs (5.25 × 10^6^/mL to 1.01 × 10^6^/mL in naïve controls). Similar to the vehicle controls, FTY720-treated animals displayed an 89% decrease in lymphocytes at 6 hrs (0.6 × 10^6^/mL), whereas lymphocytes remained reduced at 72 hrs (81% reduction, 0.99 × 10^6^/mL) which is in stark contrast with vehicle control animals. By contrast, the number of circulating neutrophils (Figure 2(c)) increased at 6 hrs (435% increase) and 24 hrs (577% increase) compared with the naive control group. In FTY720-treated rats, neutrophils increased with a peak at 6 hrs (750% higher than naïve control levels) and declined to below naïve control levels (55% reduction) at 72 hrs.

**Figure 3:**
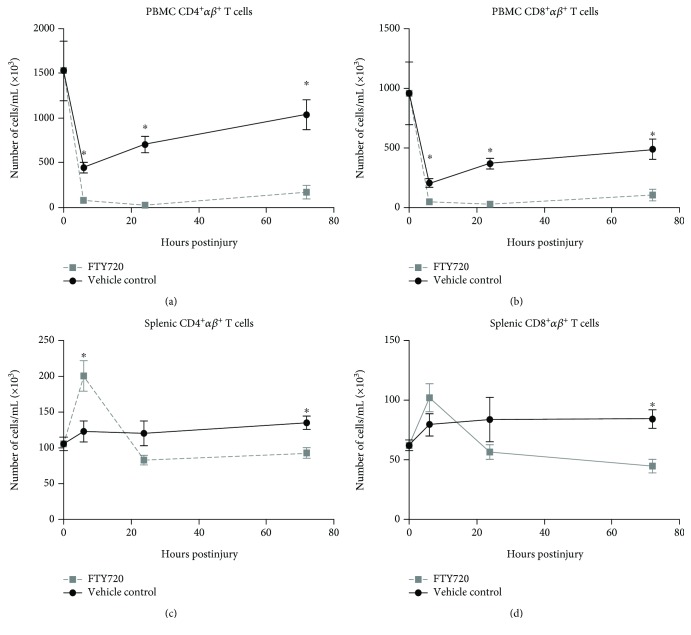
FTY720 sequesters peripheral T cells with a differential treatment effect on splenic T cell populations. Rats underwent renal ischemia by the surgical placement of a microvascular clamp around the left renal pedicle of the anesthetized rat. Rats received a single intravenous bolus of either vehicle control (black circle) or 0.3 mg/kg FTY720 (gray square) 15 min after placement of the clamp. Treatment groups were euthanized at 6, 24, or 72 hrs. Peripheral blood (a, b) and isolated splenocytes (c, d) were prepared for flow cytometry analysis by staining for T cell markers CD4, CD8, and *αβ*. Data are presented as the calculated mean ± SEM number of circulating cells per mL of blood and per spleen (*n* = 5-10 rats per time point/group). ^∗^ *p* < 0.05 compared with vehicle control at the same time point postinjury.

The percentages of circulating CD4^+^ *αβ* T cells and CD8^+^ *αβ* T cells significantly decreased in both injury groups at 6 hours vs. naïve control (Figures 3(a) and 3(b)). However, vehicle control rats displayed a trend towards recovery at 72 hours with CD4^+^ *αβ* T cells and CD8^+^ *αβ* T cells at 32% and 49% below naive controls, respectively. By comparison, FTY720-treated rats displayed reductions of 89% and 89% compared to naïve control for peripheral CD4^+^ *αβ* T cells and CD8^+^ *αβ* T cells, respectively, at 72 hours. In vehicle control rats, the 6-hour time point represents the nadir followed by recovery up to 72 hours for both T cell populations, whereas FTY720-treated rats displayed a nadir at 24 hours with minimal recovery. Importantly, peripheral CD4^+^ *αβ* and CD8^+^ *αβ* T cell populations were significantly reduced in FTY720-treated rats vs. vehicle-treated rats at each study time point (*p* < 0.05). By contrast, splenic T cell populations were increased by 28% (CD4) and 35% (CD8) in vehicle-treated rats at 72 hours postinjury. FTY720 treatment resulted in alterations in the kinetics of splenic T cells, including an increase in both T cell populations (CD4^+^ *αβ* T cell 89% increase and CD8^+^ *αβ* T cells 64% increase) at 6 hrs postinjury followed by a return to levels near naïve control values at 24 and 72 hrs. Splenic CD4^+^ *αβ* T cells were significantly increased in FTY720-treated rats vs. vehicle control rats at 6 hrs (*p* = 0.016), whereas CD8^+^ *αβ* T cells had a similar trend that was not statistically significant (*p* = 0.181).

### 3.2. FTY720 Treatment Improved Liver Damage and Clinical Features of Disease

**Figure 4:**
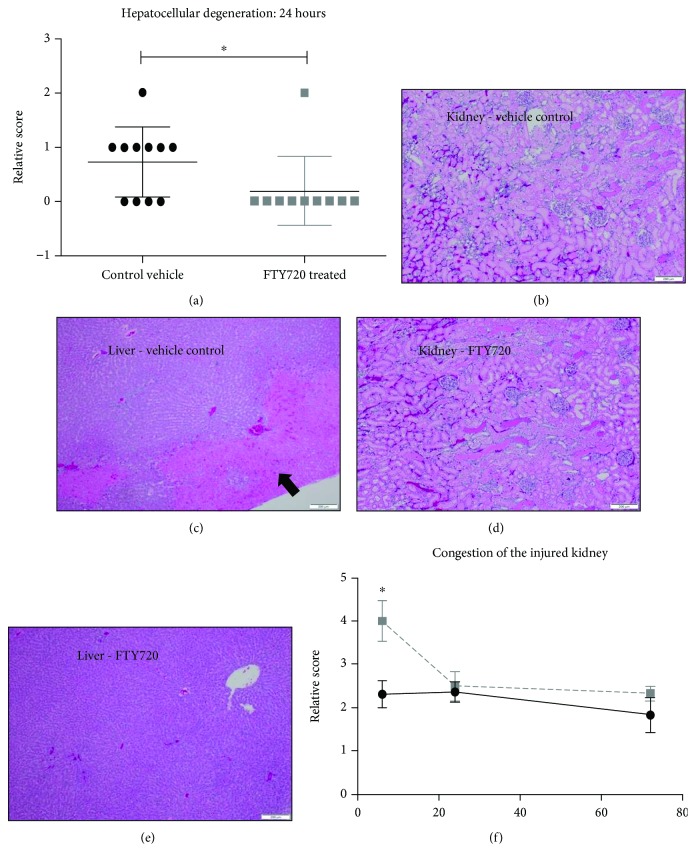
FTY720 treatment reduces hepatocellular degeneration compared to vehicle controls. Formalin-fixed paraffin-embedded liver and ischemic kidney tissue was stained with hematoxylin and eosin at 6, 24, and 72 hrs. Digital bright-field images were taken at 40x total magnification and analyzed by a pathologist using a previously established scoring system. Vehicle control (black circle) and FTY720 treatment (gray square) at the indicated time points postinjury. *p* < 0.05 compared with vehicle control at the same time point postinjury. Representative images of the liver at 24 hrs for vehicle control-treated (b) and FTY720-treated (c) rats. Arrow pointing to hepatocellular degeneration (b). Representative images of the ischemic kidney at 24 hrs for vehicle control-treated (d) and FTY720-treated (e) rats. (f) Comparison of renal congestion between vehicle control (black circle) and FTY720-treated (gray square) rats.

Vehicle-treated animals displayed mild, but measureable, hepatocellular degeneration at 24 hrs postinjury (Figure 4(a)). By contrast, FTY720 treatment demonstrated a significant reduction in hepatocellular degeneration as compared to the vehicle control group (*p* = 0.02). Representative images demonstrated the treatment effect on liver injury. Mild hepatocellular degeneration (1 out of 5) can be seen in the pink coloration denoted by the arrow (Figure 4(b)) in the vehicle control, but not in the FTY720-treated rat (Figure 4(c)). This effect was transient however as there were reduced signs of hepatocellular degeneration in the vehicle control-treated group at 72 hrs (data not shown). Ischemic kidney tissues from both vehicle control (Figure 4(d)) and FTY720 (Figure 4(e)) treatment groups displayed a high degree (5 out of 5) of tubular degeneration and necrosis. We observed that at 6 hrs postinjury, FTY720 treatment significantly increased (*p* = 0.01) renal congestion in the ischemic kidney (Figure 4(f)) as well as perivascular edema in the lungs (data not shown) when compared to the vehicle-treated control. No significant differences in renal congestion or pulmonary perivascular edema were observed at 24 or 72 hrs between treatment groups.

**Figure 5:**
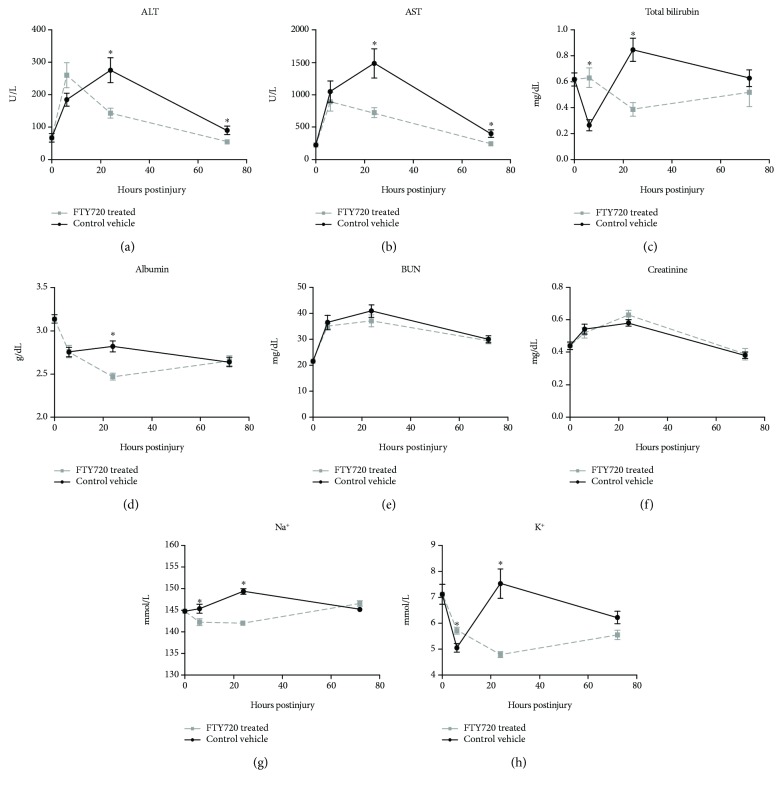
FTY720 treatment reduces serum features associated with liver injury. Data are shown as mean ± SEM serum alanine aminotransferase (ALT), aspartate aminotransferase (AST), total bilirubin, albumin, blood urea nitrogen (BUN), creatinine, Na^+^, and K^+^ levels for vehicle control (black circle, *n* = 10 rats) and FTY720-treated (gray square, *n* = 10 rats) rats at the indicated time points postinjury. Time 0 values represent data from untreated naïve rats. ^∗^ *p* < 0.05 compared with vehicle control at the same time point postinjury.

In vehicle control rats, levels of ALT (Figure 5(a)), AST (Figure 5(b)), and total bilirubin (Figure 5(c)) were measured. At 24 hrs post-IRI, concomitant with hepatocellular degeneration (Figure 4(a)), FTY720-treated rats had significantly lower mean ALT (*p* < 0.01), AST (*p* < 0.01), and total bilirubin values (*p* < 0.01) and a significantly greater degree of hypoalbuminemia (Figure 5(d); *p* < 0.01) when compared with the vehicle control group at 24 hrs. Blood urea nitrogen (BUN) and serum creatinine levels peaked at similar levels at 24 hrs in both treatment groups (Figures 5(e) and 5(f)). Serum Na^+^ and K^+^ levels each peaked at 24 hrs in vehicle control-treated animals (Figures 5(g) and 5(h)) before returning at 72 hours to levels approximately similar to those of naïve controls. Conversely, FTY720-treated animals exhibited nadirs at 24 hrs that were significantly lower than those levels observed in vehicle-treated animals for both Na^+^ (*p* = 4.09 × 10^−6^) and K^+^ (*p* < 0.01).

### 3.3. qPCR Analysis Indicated Increased Chemokine and Inflammatory Cytokine Expression in Vehicle-Treated Rats vs. FTY720-Treated Rats at 6 hrs Postinjury

**Figure 6:**
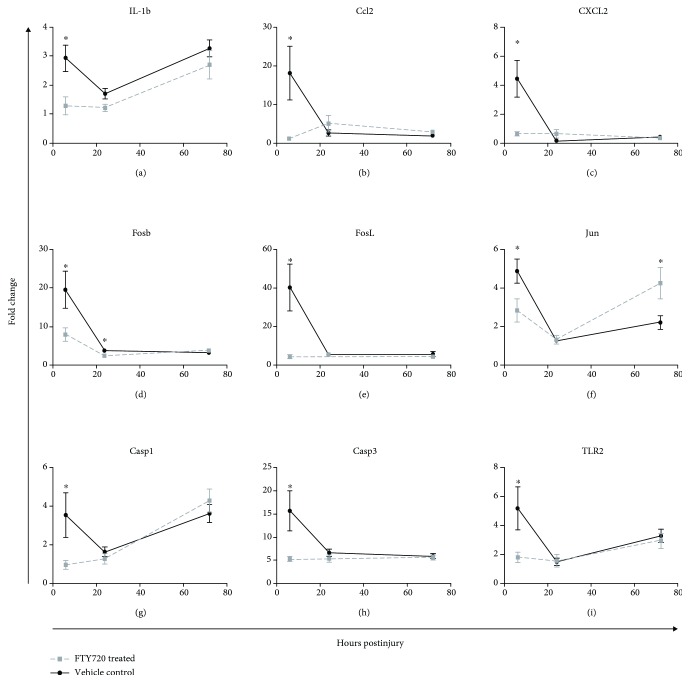
Differential expression of the select gene in the liver in FTY720-treated rats (gray square) versus vehicle control-treated rats (black circle) following renal ischemia. Tissues at the indicated time point post-IRI were stored in RNAlater and subsequently processed for mRNA. cDNA conversion of 1 *μ*g of RNA was performed by RT-PCR followed by semiquantitative real-time PCR for gene expression analysis using the 2^-ΔΔCt^ method. A custom low-density array panel of 88 genes relevant to ischemia-reperfusion injury was selected for the analysis. Gene expression was quantified relative to liver tissue from naïve control animals. *p* < 0.05 compared with vehicle control at the same time point postinjury.

**Figure 7:**
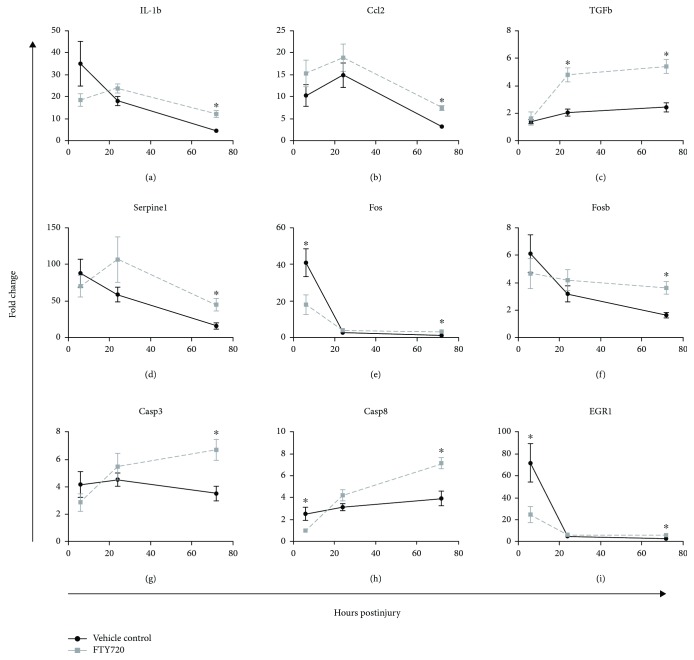
Differential expression of the select gene in the ischemic kidney in FTY720-treated rats (gray square) versus vehicle control-treated rats (black circle) following renal ischemia. Tissues at the indicated time point post-IRI were stored in RNAlater and subsequently processed for mRNA. cDNA conversion of 1 *μ*g of RNA was performed by RT-PCR followed by semiquantitative real-time PCR for gene expression analysis using the 2^-ΔΔCt^ method. A custom low-density array panel of 88 genes relevant to ischemia-reperfusion injury was selected for the analysis. Gene expression was quantified relative to kidney tissue from naïve control animals. *p* < 0.05 compared with vehicle control at the same time point postinjury.

Differentially expressed transcripts for IRI-associated gene targets were detected in the ischemic kidney (Figure S1A), contralateral kidney (Figure S1B), lung (Figure S1C), and liver (Figure S1D), which are displayed in the supplemental figure as heat maps. Based on the patterns of gene expression displayed here, select genes were chosen for display from the liver (Figure 6) and ischemic kidney (Figure 7).

The expression of multiple genes in the liver was significantly decreased in rats receiving FTY720 vs. vehicle control-treated rats at 6 hrs. This includes prototypical inflammatory cytokine IL-1*β* (Figure 6(a)), chemokines such as CCl2 and CXCL2 (Figures 6(b) and 6(c)), and AP-1 components Fosb (Figure 6(d)), FosL1 (Figure 6(e)), and Jun (Figure 6(f)), as well as apoptotic genes Casp1 (Figure 6(g)), Casp3 (Figure 6(h)), and Casp8 (data not shown). Notably, the treatment effect is primarily observed at 6 hrs with both injury groups displaying comparable expression levels in genes of interest at 24 and 72 hrs.

Gene expression in the ischemic kidney followed a different pattern from that observed in the liver. Both FTY720-treated and vehicle control animals displayed increases in gene expression associated with inflammation such as IL-1*β* and chemokine CCl2 (Figures 7(a) and 7(b)). No treatment effect was observed for these genes at 6 hrs or 24 hrs. Rather, FTY720 treatment was associated with significantly increased expression of several inflammatory cytokines at 72 hrs, including IL-1*β* and CCl2 and AP-1 components (Fos and Fosb), as well as proapoptotic genes Casp3 (Figure 7(g)) and Casp8 (Figure 7(h)). Additionally, FTY720 treatment was associated with an increase in expression of the regulatory gene TGF*β* at 24 and 72 hrs postinjury. An exception to this pattern was EGR1 (Figure 7(i)) which was upregulated 24.8-fold in FTY720-treated animals vs. 71.8-fold in vehicle control animals at 6 hrs, with expression decreasing in both treatment groups at 24 and 72 hrs.

## 4. Discussion

Renal IRI initiates a complex and dysregulated immune response that mediates damage to peripheral organs, with potentially significant sequelae such as fulminant multiorgan failure (MOF) [2, 23]. The local inflammatory response generated by hypoxic stress during ischemia promotes systemic inflammation following reperfusion of the ischemic kidney(s). As such, mitigating the severity of this inflammatory response is a potential strategy for limiting or preventing MOF and improving long-term patient outcomes. In addition to the strength of the inflammatory response, the time between ischemic insult and repair/reperfusion is critical. Prolonged periods of renal ischemia can result from low flow states (sepsis, hemorrhage), abdominal compartment syndrome, direct renal trauma, arterial or venous thrombosis, or embolic disease. Early intervention in ischemic injury as a means of mitigating or alleviating the severity of IRI has received considerable attention. “Golden hour” management following traumatic injury has long been held as a critical window for therapeutic intervention [24]. However, the time between traumatic injury and hospital surgical resuscitation is highly variable and can be prolonged in both civilian and military trauma. The risk of death increases directly with increased time to treatment, even within the first 30 min following traumatic injury to the torso [24]. Novel treatments that can be administered prior to hospitalization may be beneficial in reducing morbidity and mortality in these patients. One such candidate, FTY720, a drug currently approved for the treatment of multiple sclerosis, has shown promise. In this experimental study, we observed the protective effects of a single FTY720 infusion on liver injury that occurred subsequent to renal IRI.

FTY720 did not demonstrate a beneficial effect in the (local) ischemic renal tissue but rather was effective in reducing a secondary injury in the liver. This was demonstrated both by a significant reduction in hepatocellular degeneration at 24 hrs and by serum markers of liver injury and function with respect to the vehicle control group. Amelioration of liver injury due to FTY720 treatment in this renal ischemia reperfusion injury model was associated with a reduction in circulating CD4^+^ T cells as seen in prior studies [25]. However, FTY720 has also been shown to mitigate the severity of liver injury by reducing the number of infiltrating macrophages in a murine model of hepatitis [26]. While infiltrating macrophages have a well-established role in inflammation, the literature strongly supports a role for CD4^+^ in the pathogenesis of IRI [9–11, 27].

In support of this postulation, gene expression data indicated several trends of interest. Analysis of mRNA isolated from liver tissue revealed a trend of increased expression of inflammatory markers, apoptosis-associated genes (Casp1, Casp3, and Casp8), AP-1 components, and genes associated with hypoxic stress (Hmox1, TLRs, Serpine1, etc.) in vehicle control vs. FTY720-treated groups. Given that these changes occurred prior to the observed improvement in hepatocellular degeneration, it is possible that FTY720 reduces injury to the liver by reducing inflammation. This conclusion is supported by a reduction in the expression of chemokines, including CCL2 and CXCL2 at 6 hrs in FTY720-treated animals, concomitant with the sequestration of T cells from the periphery.

Finally, it is notable that EGR-1 gene expression is approximately 3- to 4-fold higher in vehicle-treated vs. FTY720-treated ischemic kidney tissue. It has been suggested that EGR-1 is a “master regulator” of gene expression following ischemic stress [28]. However, it is not clear from these findings how differential expression of EGR1 in the ischemic kidney, which did not display a treatment effect with respect to histopathology, would affect liver pathology at 24 hrs.

The findings presented here with respect to the effect of FTY720 on local ischemic tissue are in contrast to previous findings in a rat model of IRI [29, 30]. Notably, it has been demonstrated that the oral application of FTY720 6-24 hours prior to surgery was effective in mitigating local injury to ischemic kidney tissue. While these findings are apparently confounding to those presented here, several critical differences in experimental design may account for the divergent results. First, Dragun et al. used a model of kidney transplantation wherein kidneys are excised from donor animals, cold preserved in a solution for 4 hours, and then transplanted into a recipient rat [29]. In contrast, Delbridge et al. used a different model of renal IRI wherein the right kidney was surgically removed while the left kidney was clamped, as done here, for approximately 60 minutes. Rats euthanized at 3 days demonstrated reduced apoptosis in the ischemic kidney [30]. These observations likewise conflict with the data presented here, that is, data of increased apoptotic gene expression in renal tissue with FT720 treatment at 72 hours. In contrast to Dragun et al., the model presented here uses a surgically placed clamp to stop blood flow in situ with subsequent reperfusion. Therefore, local ischemic tissues do not benefit from cold preservation during the period of ischemia. Both studies rely on pretreatment with FTY720, which may better prepare the affected renal tissue to hypoxic conditions. While potentially relevant to the transplantation setting, it must be stressed that the intentions of this study were to replicate renal IRI resulting from traumatic injury rather than from transplantation. In this regard, it is difficult to directly compare results of pretreatment with FTY720 to those of postinjury treatment as performed here. Thus, FTY720 treatment was given shortly after injury, rather than prior to clamp placement to mimic a clinical scenario for treatment in a trauma setting. Additionally, the ischemic time in the current model was much longer than previous experimental designs and may contribute to the nonprotective effects on the ischemic kidney. Furthermore, there are critical genetic differences between Dragun et al. which used the Lewis rat and this study which used the Sprague-Dawley rat. Notably, the Lewis rat has been demonstrated to be more resistant to ischemic injury than the Sprague-Dawley rat [31]. As such, critical differences in experimental design likely contribute to the divergent results observed here as compared with previous findings.

Our data suggest that the degree of kidney injury was unaffected by FTY720 treatment, despite improvements in peripheral organ damage caused by reperfusion injury. The contrast between ischemic and reperfusion injury in this study suggests both a value and possible limitation of using FTY720. That is, treatment most likely benefits peripheral tissues by reducing the inflammatory insult to distant organs but does not benefit local tissues primarily affected by ischemic insult. This statement contrasts with the findings that total T cell depletion (using SCID animals) results in mitigated injury to the site of ischemia [11]. The reason for this disparity may relate to the degree of T cell depletion, which is transient and localized with FTY720 treatment compared with complete depletion in a genetic knockout model. Alternatively, it may be that multiple FTY720 treatments and/or increased dosage may further improve disease outcome in this model. Another possible explanation is that T cells in the hypoxic (renal) compartment are not exposed to FTY720 until after reperfusion due to the microvascular clamp that restricted blood flow. It is important to indicate however that the data presented here do not explicitly implicate a direct role for T cells in mediating either kidney or liver injury in this model. It is possible that the benefits of FTY720 presented here are mediated through a different leukocyte subset. However, it is consistent with the existing literature which highlights a direct role for T cells in mediating injury in rodent models of renal IRI [10–13]. Additionally, the literature referenced here identify a role for T cells in mediating a local injury using models of renal IRI which are most relevant to trauma-induced renal IRI. Further testing is necessary however both to confirm the role of T cells in this model and to better understand why the liver benefits from FTY720 treatment while local ischemic tissues do not.

Our histopathology scores indicated potential adverse effects of FTY720 as demonstrated by increased renal congestion in the ischemic kidney and perivascular edema in the lungs. However, serum indicators of kidney function were not significantly altered at any time point due to treatment. The negative effects of FTY720 on the kidney or lung were transient, with both vehicle- and FTY720-treated groups presenting similar scores at 24 and 72 hrs. Notably, there is a strong trend towards a decrease in gene expression in the lungs due to FTY720 treatment at 72 hours for many of the genes tested (Supplementary Figure 1C). However, high in-group variability precluded statistical significance. Additional testing is required to confirm this trend and to determine its relevance. While this may be indicative of a positive treatment effect in the lungs, repeated tests as well as later time points (after 72 hours) are needed. Similarly, gene expression profiles for the ischemic kidney (Supplementary Figure 1) do not indicate changes in the expression of inflammatory or proapoptotic genes. Long-term studies are necessary to confirm a physiological improvement with treatment as well as to confirm the presence of potentially negative side effects.

## 5. Conclusion

Using a clinically relevant rat model of renal IRI, we observed a significant improvement in secondary organ injury due to FTY720 treatment that was reflected in histopathology, serum chemistry, gene expression, and CBC analysis. These findings, combined with the known mechanism of action of FTY720 as a lymphocyte-sequestering agent, are consistent with previous findings highlighting the role of lymphocytes in mediating IRI. In this study, lymphocyte sequestration was associated with reductions in the expression of proinflammatory and proapoptotic genes in the liver, which preceded reductions in serum liver enzymes and reduced histopathological signs of injury. Taken together, these observations support our hypothesis that FTY720 may improve disease outcome by modulating the immune response associated with renal IRI. FTY720 as an initial treatment following traumatic injury involving ischemia-reperfusion injury may attenuate secondary end-organ damage and potentially the long-term sequelae of this pathophysiologic insult.

## Figures and Tables

**Table 1:** Tissue sections, stained with hematoxylin and eosin, were assessed using an established scoring system. Lesions observed within the section were graded by the percentage of the section affected by the lesion as well as by the number of foci observed as follows.

Grading scheme for focal and multifocal lesions			
Severity	Percent of the sections affected	Grade	Quantifiable finding
Normal	0%	0	No foci
Minimal	<5%	1	1-2 foci
Mild	5-25%	2	3-6 foci
Moderate	25-50%	3	7-12 foci
Marked	50-75%	4	>12 foci
Severe	75-100%	5	Diffuse

**Table 2:** Lung tissue sections, stained with hematoxylin and eosin, were assessed using an established scoring system. Areas of pulmonary perivascular edema were scored based on the observed width of the arterial wall relative to the average width in uninjured tissues. A grading system describing the severity of edema was linked to the categorical scoring system based on this assessment as follows (see also Figure 1).

Grading scheme for pulmonary perivascular edema			
Severity	Extent of edema	Grade	Quantifiable finding
Normal	None	0	None
Minimal	Very small amount	1	Up to 2 times the average width of the arterial wall
Mild	Small amount	2	3 to 5 times the average width of the arterial wall
Moderate	Moderate amount	3	6 to 7 times the average width of the arterial wall
Marked	Large amount	4	8 to 10 times the average width of the arterial wall
Severe	Very large amount	5	>10 times the average width of the arterial wall

## Data Availability

The data used to support the findings of this study are available from the corresponding author upon request.

## Supplementary Materials

Supplementary Materials Supplemental Figure S1: Differential expression heat maps from tissue in FTY720-treated versus vehicle control-treated rats following renal ischemia.
