# Supplementary material for: FTY720 Effects on Inflammation and Liver Damage in a Rat Model of Renal Ischemia-Reperfusion Injury
Source: Mediators Inflamm. 2019 Mar 20;2019:3496836. doi: 10.1155/2019/3496836 (PMC6446098; doi:10.1155/2019/3496836)

Supplemental Figure S1: Differential expression heat maps from tissue in FTY720-treated versus vehicle control treated rats following renal ischemia. Tissues collected at 6, 24, and 72 hr point post IRI were stored in RNALater and subsequently processed for mRNA. cDNA conversion of 1µg of RNA was performed by RT-PCR followed by semi-quantitative real-time PCR for gene expression analysis using the $2^{-\Delta\Delta CT}$ method. Data was then transposed into a Log_2 Base format to better display the range of expression observed. A custom low density array panel of genes relevant to ischemia reperfusion injury was selected for the analysis. For each tissue, gene expression was quantified relative to naïve control tissue.

Supplemental Figure S1-A:

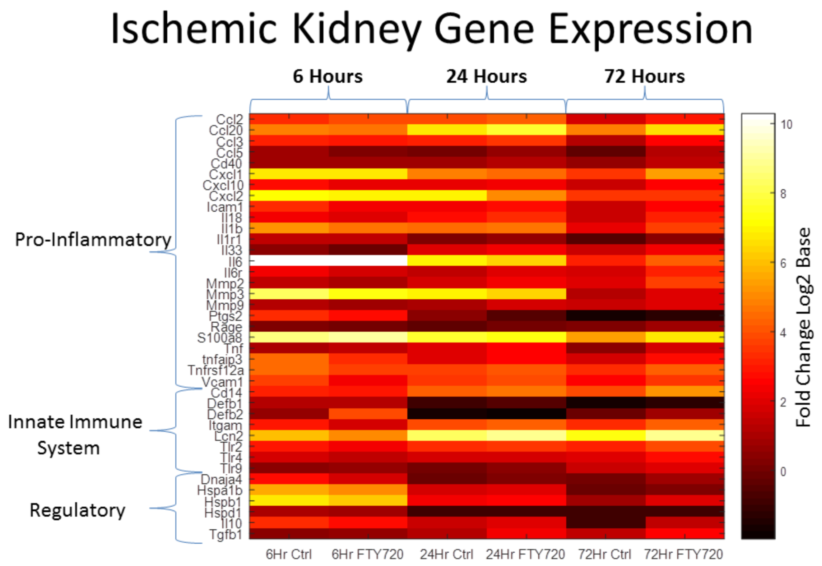

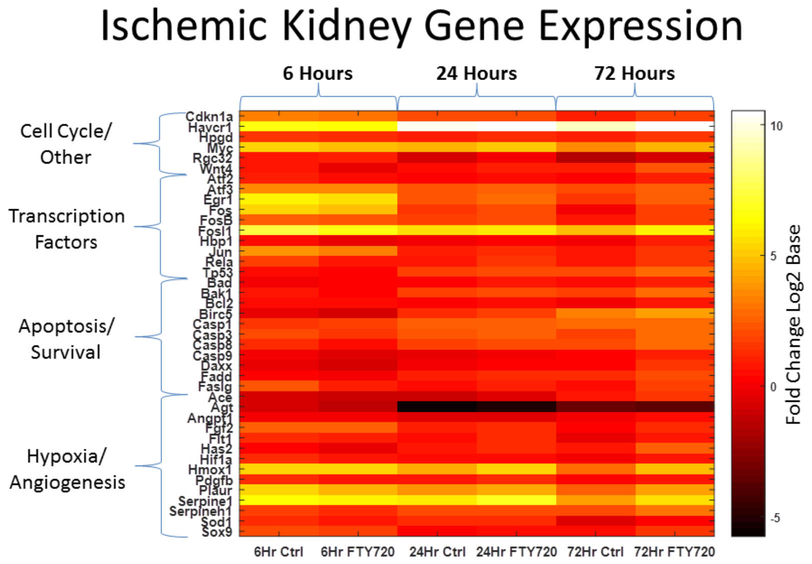

Supplemental Figure S1-B:

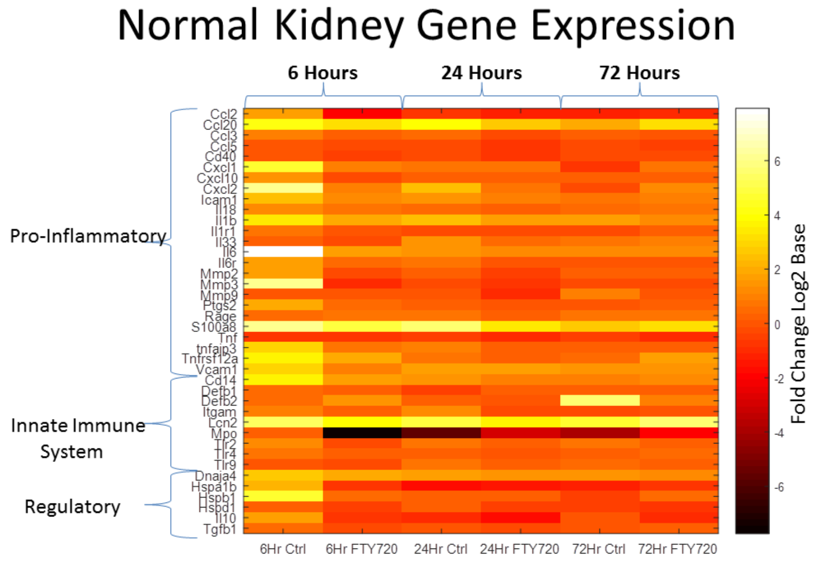

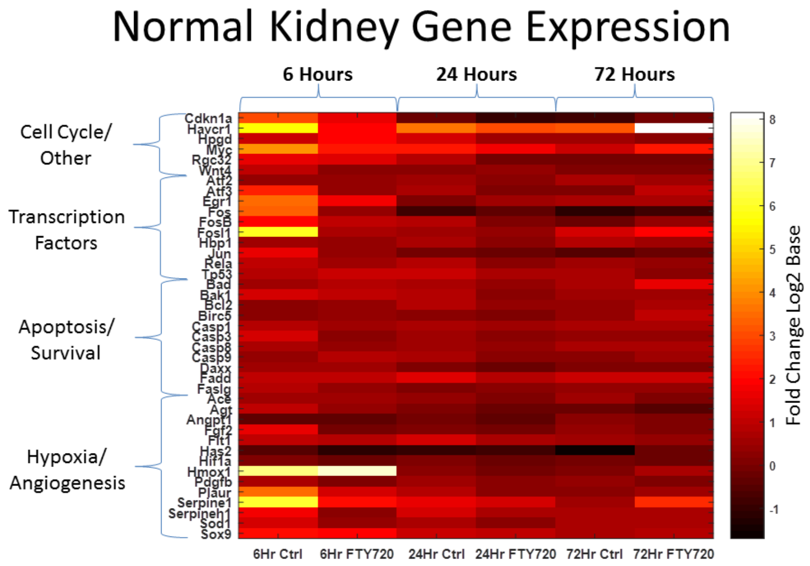

Supplemental Figure S1-C:

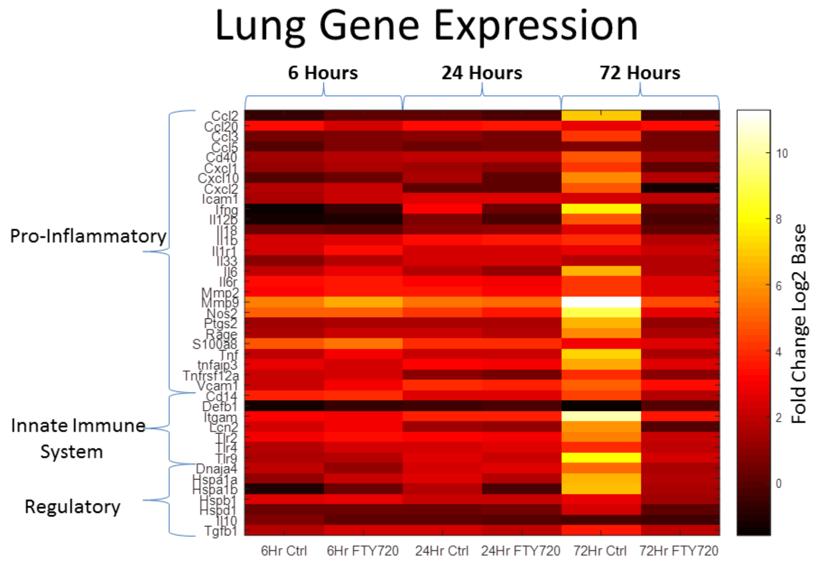

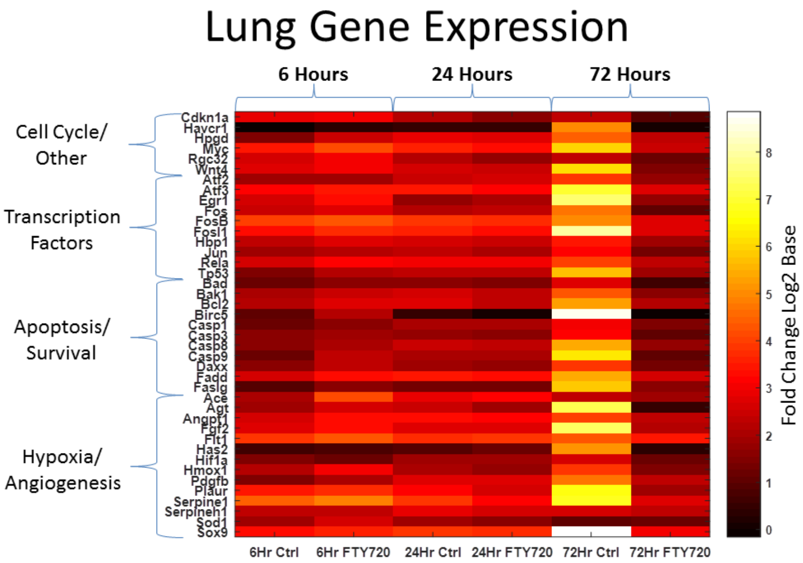

Supplemental Figure S1-D:

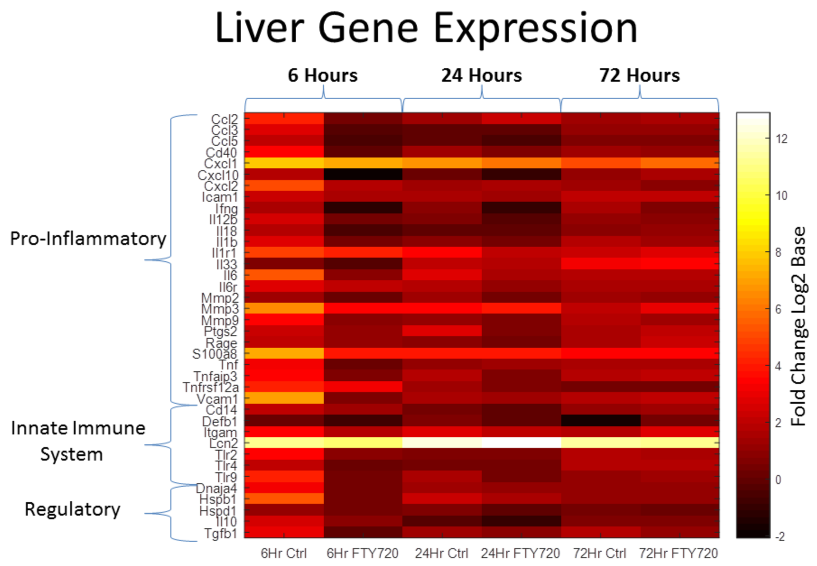

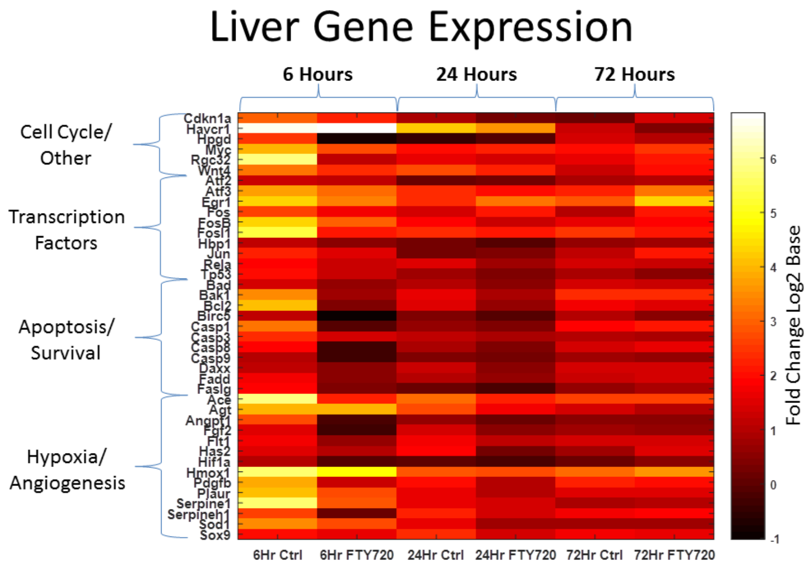

Supplement: Supplementary Materials — Supplemental Figure S1: Differential expression heat maps from tissue in FTY720-treated versus vehicle control-treated rats following renal ischemia. [file 3496836.f1.pdf]
